# Fc‐null anti‐CTLA‐4 antibody: a novel strategy to facilitate cancer immunotherapy by ridding the colitis‐inducing mishap

**DOI:** 10.1002/mco2.622

**Published:** 2024-06-14

**Authors:** Jingjing Chen, Dexuan Wang, Hu Zhang

**Affiliations:** ^1^ Department of Gastroenterology West China Hospital Sichuan University Chengdu China; ^2^ Department of Pediatrics The Second Affiliated Hospital and Yuying Children's Hospital of Wenzhou Medical University Wenzhou China

## Abstract

Writing recently in Science, Lo and coworkers characterized a critical role of the gut microbiota in CTLA‐4 blockade‐induced colitis, revealing that an Fc domain deficient anti‐CTLA‐4 antibody can elicit antitumor responses effectively while avoiding the induction of colitis‐like disease.1 This research opens up novel avenues for employing anti‐CTLA‐4 antibody therapy to circumvent the onset of colitis, which is often considered the Achilles' heel of what is arguably the most efficacious treatment for certain blood cancers and/or solid tumors.

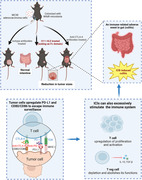

1

Writing recently in *Science*, Lo and coworkers characterized a critical role of the gut microbiota in CTLA‐4 blockade‐induced colitis, revealing that an Fc domain‐deficient anti‐CTLA‐4 antibody can elicit antitumor responses effectively while avoiding the induction of colitis‐like disease.^1^ This research opens up novel avenues for employing anti‐CTLA‐4 antibody therapy to circumvent the onset of colitis, which is often considered the Achilles' heel of what is arguably the most efficacious treatment for certain blood cancers and/or solid tumors.

Immune‐checkpoint inhibitors (ICIs), involving anti‐CTLA‐4 and anti‐PD‐1 antibodies, have emerged as effective cancer treatments over the past decade. The anti‐CTLA‐4 antibodies, such as ipilimumab and tremelimumab, are widely applied as therapeutic agents in clinical trials of different cancers. These therapeutics enhance antitumor immune responses by blocking negative signals to trigger T‐cell activation, thus restoring tumor surveillance and effectively destroying tumor cells. However, these immunotherapies can also induce inflammatory toxicities known as immune‐related adverse events (irAEs), which are considered off‐target effects resulting from excessively activated immune responses. Immune checkpoint blockade (ICB)‐induced colitis represents one of the most frequent and severe irAEs among patients treated with anti‐CTLA‐4 antibodies, either alone or in combination with anti‐PD‐1 antibodies. This condition cuts the therapy short and reduces the quality of life, ultimately causing fatality. Limited mechanistic understanding of the ICB‐induced colitis impedes continuous application of the therapeutics and finding approaches to alleviate the tissue damage associated with the therapy. CTLA‐4 inhibition may trigger intestinal inflammation that affects the entire colon rather than one segment of it, leading to severely compromised mucosa with diffuse ulceration and edema, as observed in the endoscopic examination.^2^ Additionally, ICB‐induced colitis is characterized by an increased presence of CD4^+^ effector and Tregs cells, along with enrichment of CD8^+^ tissue resident memory T (TRM) cells.^3^ Furthermore, CTLA‐4 blockade may lead to a loss of self‐tolerance to mucosal flora and autoantigens. Depletion of Tregs cells in the intestinal mucosa appears to play a critical role in this process, though not all studies can capitulate Tregs depletion following anti‐CTLA‐4 treatment.^4^ Consequently, it is necessary to carry out mechanistic science, and develop alternative treatments to circumvent ICB‐induced colitis without compromising the benefits of antitumor immunity (Figure 1).

**FIGURE 1:**
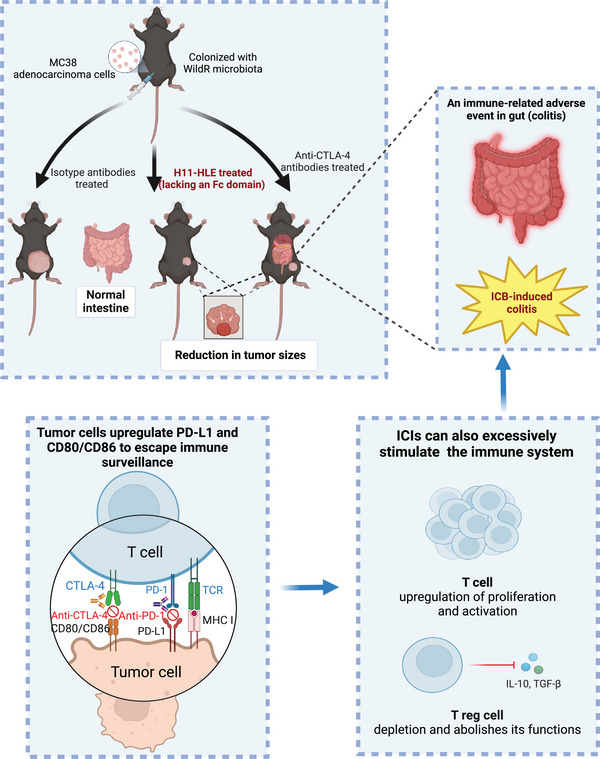
Fc‐null anti‐CTLA‐4 antibody has similar antitumor immunity‐promoting effects and does not induce colitis in mice harboring WildR microbiota, who are otherwise susceptible to colitis when receiving conventional anti‐CTLA‐4 antibodies. Immune checkpoint inhibitors can strongly enhance the immune function; therefore, immune cells widely spread infiltrate into different organs, leading to diverse immune‐related adverse events, such as colitis in the lower digestive tract. The upper part is a brief overview of the whole experiment of this study, and the lower two panels are designed to explain the way of tumor cells escaping from immune surveillance and the underlying mechanism of the ICB‐induced colitis, respectively. WildR: wild mouse microbiome reconstituted; ICB: immune checkpoint blockade; H11‐HLE: CTLA‐4‐binding H11 VHH with a half‐life extender.

Built on observations of ICB‐induced colitis in free‐living mice harboring wild microbiota, Lo et al. performed a functional comparison of antibodies directed against CTLA‐4 or PD‐1 in the onset of ICB‐driven gut inflammation.^1^ In the cecal immune cells, an accumulation of IFNγ^+^ or IL‐17^+^ T helper (T_H_) cells and dual‐positive IFNγ^+^IL‐17^+^CD4^+^ T_H_ cells was noted by Day 9 after CTLA‐4 blockade. Additionally, strong Tbet but comparatively moderate RORγt expression in gut CD4^+^ T cells underscores a T_H_1‐skewed response during ICB‐driven inflammation. The proportion of Foxp3^+^ T cells decreases, along with a selective depletion of RORγt^+^ Tregs throughout the ICB treatment period, indicating that CTLA‐4 inhibition preferentially induces a T_H_1 response and a biased depletion of RORγt^+^ pTregs in the gut. Findings from selective depletion of CD4^+^ and CD8^+^ T cells or the use of IFNγ‐neutralizing antibodies in ICB‐driven colitis indicated that CD4^+^ T‐cell‐mediated responses and IFNγ are primarily responsible for the intestinal inflammation caused by CTLA‐4 blockade. Moreover, data from single‐cell RNA sequencing (scRNA‐seq) affirmed that maintaining nonthymic Tregs is closely linked to intestinal homeostasis during ICB treatment.

Considering the above discovery that CTLA‐4 blockade induces intestinal inflammation via CD4^+^ T cells and IFNγ, the authors further explored the pathogenic roles of CD4^+^ T cells. Following treatment with anti‐CTLA‐4 antibody, a significant increase in IFNγ^+^ Tbet^+^ T_H_1 cells and a selective decrease in pTregs were noted. This suggests that under homeostatic conditions, colitic T_H_1 cells are generally restricted, but can be specifically activated by CTLA‐4 inhibitors when accompanied with wild mouse microbiome‐reconstituted (WildR) mice.

Numerous preclinical and clinical studies have indicated that FcRγ‐dependent depletion of Tregs contributes to the antitumor efficacy induced by ICB.^4^ The authors observed minimal fecal LCN‐2 induction, absence of immunopathology in cecal tissues, and low/no cytokine production by CD4^+^ T cells in FcRγ‐deficient mice colonized with WildR microbiota. Therefore, Fc domain‐dependent functions of anti‐CTLA‐4 antibodies may be essential for gut inflammation, as evidenced in a humanized mouse model of CTLA‐4 blockade.

To investigate the necessity of Fc‐FcγR interactions in ICB‐induced colitis, the authors developed an extended half‐life H11 (H11‐HLE) antibody, comprised solely of heavy chain fragments (VHHs) targeting CTLA‐4. They used mice colonized with WildR microbiota and inoculated with tumor cells to simultaneously monitor ICB‐mediated tumor rejection and colitis. When administered with monotherapeutic checkpoint blockade, tumor‐inoculated mice treated with H11‐HLE exhibited comparable antitumor activity to those treated with anti‐CTLA‐4 antibodies, yet without the induction of colitis. A decreased percentage of Foxp3^+^ Tregs in CD4^+^ T cells was observed in animals treated with either anti‐CTLA‐4 antibodies or H11‐HLE compared to the isotype control, albeit to a lesser extent. These observations suggest that Fc domain‐deficient nanobodies targeting CTLA‐4 can still trigger strong antitumor responses without causing significant gut irAEs. Additionally, mice treated with H11‐HLE alongside anti‐PD‐1 or anti‐PD‐L1 antibodies did not develop colitis. Their work provides critical evidence for the utility of an Fc domain‐lacking anti‐CTLA‐4 antibody, either alone or combined with anti‐PD‐1 or anti‐PD‐L1 antibodies in effectively stimulated antitumor responses, but not causing colitis, presenting potential benefits to the suffering cancer patients in clinical settings (Figure 1).

Several early studies indicated that CTLA‐4 could restrain T‐cell activation independently of Tregs in both autoimmunity and antitumor responses.^5^ Hence, further researches are required to thoroughly examine the cumulative effects and the underlying mechanisms of T‐cell CTLA‐4 inhibition and the depletion of Tregs by anti‐CTLA‐4 antibodies during colitis. Notably, ICIs including anti‐CTLA‐4 antibodies can lead to irAEs across a broad range of organ systems, manifesting with diverse frequencies and severities. Thus, investigating other types of irAEs, including cutaneous irAEs, pulmonary irAEs, cardiac irAEs, neurological irAEs, and so forth,^2^ is equally important. Moreover, the potential of utilizing Fc domain‐removed antibodies in conjunction with other immune checkpoint inhibitors merits further exploration. Considering the divergence between human and mouse models, ICB‐induced irAEs in humans may not be entirely replicated in animal models. Therefore, researchers must be prudent in interpreting results from animal studies, and there is a crucial need for their validation in primates and humans before translating the research into bench‐side. Setting a stepping stone with this encouraging study, scientists in the related field may be better prepared to develop next‐generation CTLA‐4 inhibitors with reduced inflammatory toxicities, and thereby improved therapeutic potency.

## AUTHOR CONTRIBUTIONS

**Jingjing Chen** wrote the initial draft of the manuscript and drew the figure. **Dexuan Wang** and **Hu Zhang** supervised and revised the manuscript. All authors have reviewed and approved the article.

## CONFLICT OF INTEREST STATEMENT

The authors declare they have no conflicts of interest.

## ETHICS STATEMENT

Not applicable.

## Data Availability

Not applicable.
